# Child Maltreatment Disclosure to a Text Messaging–Based Crisis Service: Content Analysis

**DOI:** 10.2196/11306

**Published:** 2019-03-25

**Authors:** Laura Schwab-Reese, Nitya Kanuri, Scottye Cash

**Affiliations:** 1 Department of Health and Kinesiology Purdue University West Lafayette, IN United States; 2 Crisis Text Line New York, NY United States; 3 Yale School of Management Yale University New Haven, CT United States; 4 College of Social Work The Ohio State University Columbus, OH United States

**Keywords:** child maltreatment, disclosure, SMS, text message

## Abstract

**Background:**

Disclosure is a difficult but important process for victims of child maltreatment. There is limited research on child maltreatment disclosure. Young people have been reluctant to disclose victimization to adults, but short message service (SMS) crisis services may represent one novel method of engaging young people around sensitive topics.

**Objective:**

The purpose of this study was to determine characteristics of child maltreatment disclosure to an SMS-based crisis service.

**Methods:**

We conducted a content analysis of all conversations (N=244) that resulted in a mandatory report by an SMS-based crisis service between October 2015 and July 2017. We coded characteristics of the disclosure process, including the reason for initial contact, phrase used to disclose abuse, perpetrator, type of abuse, and length of victimization. After identifying terms used by young people to disclose child abuse, we randomly selected and analyzed 50 conversations using those terms to determine if use of the terms differed between conversations that did and did not result in mandatory report.

**Results:**

Parents were the most common perpetrator. Physical abuse was the most common form of abuse discussed in the initial abuse disclosure (106/244, 43.4%), followed by psychological abuse (83/244, 34.0%), sexual abuse (38/244, 15.6%), and neglect (15/244, 6.1%). More than half of the texters discussed abuse or other significant family issues in the first message. An explicit description of the experience or definite language, such as abuse, rape, and molested, was common in disclosures.

**Conclusions:**

Early disclosure, combined with explicit language, may suggest at least a portion of young victims are actively seeking safe ways to talk about their experiences with abuse, rather than incidentally sharing experiences while seeking support for other issues. SMS text messaging may be a valuable way to engage with young people around sensitive topics, but these approaches will require careful consideration in their development, implementation, and evaluation to ensure a positive experience for young people.

## Introduction

Disclosure of child maltreatment is often a grueling experience for children and may result in the young victim re-experiencing the trauma [1]. Children and young people who disclose their experiences risk inadequate support through the disclosure process, social stigmatization associated with maltreatment victimization, retribution by the perpetrator, and removal of the victim or perpetrator from the home [1,2]. Relatively few victims disclose their maltreatment experiences to adults due to these risks [3-5]; however, disclosure is often necessary to end the abuse and connect children with resources to support their physical and psychological health [6,7]. Despite the importance of disclosure, research on children’s disclosure of physical or emotional abuse and neglect is limited [3].

Phone-based crisis lines have historically been available for young people to discretely seek support and referral to resources, but these services have not been well-adopted by young people [8]. As a result, some organizations have explored other approaches, such as short message service (SMS) text messaging, to provide crisis services to young people through their preferred methods of communication [9]. Crisis Text Line (CTL) was among the first to provide a free, 24/7 SMS line for people in crisis in the United States [10]. Since launching in August 2013, CTL’s volunteer crisis counselors have served hundreds of thousands of texters seeking help for suicidal behavior, bullying, abuse, and other crises. Other services, such as the 24/7/365 Crisis Hotline, Teens Helping Teens, and Mind Infoline, also provide SMS-based crisis services, although most provide service during limited hours or about limited topics [11-13].

Considering the significant mental health consequences of maltreatment [14], it is likely that many users of crisis services have experienced child maltreatment. To date, there have been no studies of child maltreatment disclosure using SMS-based technology. To address this gap, we conducted a content analysis of conversations between crisis counselors and texters to determine characteristics of child maltreatment disclosure to an SMS-based crisis service. The findings of this study have important implications for the use of technology to support disclosure of child maltreatment.

## Methods

### Procedures and Sample

The SMS-based crisis service provided deidentified transcripts of all conversations that resulted in a mandatory report between October 2015 and July 2017. Within this crisis service platform, conversations began when texters initiated contact and ended when texters actively ended the conversation or did not respond for an extended period. Because millions of messages have been exchanged through this service, it was not feasible to conduct content analysis with the full sample. Limiting the content analysis to conversations that resulted in mandatory report created a sample of conversations that were confirmed by crisis counselors to be about child maltreatment. Mandatory reporting is the legal requirement in the United States for professionals to report suspected child abuse or neglect to the authorities. The guidelines for mandatory reporting vary from state to state, but in most states “a report must be made when the child is known or suspected of being a victim of abuse or neglect” [15], meaning situations where the child is suspected to be in danger are required to be reported. Mandatory reporters are not required to report cases where the child has permanently left the dangerous situation or cases where adults report past childhood abuse or neglect.

However, mandatory reporting in this crisis service is complicated because user information is anonymized by the system. After disclosure of experiences consistent with maltreatment, crisis counselors are trained to disclose their status as mandatory reporters and to explicitly state that they must make a report to authorities if they know about abuse or neglect of a minor and have sufficient identifiable information (eg, name and address) to make a report. As a result, the sample does not include all instances of child maltreatment disclosure.

The Colorado Multiple Institutional Review Board determined that the study was exempt from review because no identifiable information of texters was available to researchers.

### Coding and Analysis

The research team conducted an inductive content analysis. One member of the research team read through the 244 mandatory report conversations twice to achieve immersion. During the second read, she took notes on emerging themes. Based on this process, she developed the content analysis coding framework that was revised by the research team and employees of the crisis service. Once the coding framework and data dictionary were complete, a research team member applied the coding framework (see Table 1) to the 24,730 text messages. She recoded five conversations at the mid- and endpoint of data coding—10 total—to assess coding reliability, which was greater than 95%.

All open-response sections of the coding framework (eg, language used in disclosure) were recorded verbatim, including all spelling and grammatical errors, and analyzed qualitatively following established content methods and reflexive team analysis [16-18]. Through multiple readings, the team identified themes and codes for each open-response section of the conversation. After discussing the themes and codes with the team, a research team member applied the final coding schema, which is reported in the Results section, to the open-response section of the conversations.

During the coding process, the research team recognized that several terms were frequently used to disclose maltreatment experiences. To determine if this type of language was commonly used by texters to describe experiences that was not child abuse, the team repeated the content analysis with 50 randomly selected conversations that included any of the terms commonly used during disclosure (ie, *abuse, abused, abusive*, and *molested*) from the overall pool of messages exchanged within the platform.

**Table 1:** Coding scheme of conversations that resulted in a mandatory report.

Variable	Definition	Coding approach
Year	Year of conversation from administrative system	Numerical
Month	Month of conversation from administrative system	Numerical
Age	Self-reported age of texter	Numerical
Sex or gender	Self-reported sex or gender of the texter	Categorical (*male; female; other*)
First contact	Initial text from texter	Open response
Disclosure text	First phrase and/or sentence disclosing abuse by texter	Open response
Perpetrator	Identification of perpetrator by texter	Multiple-selection categorical (*mother; father; brother; sister; stepfather/mother’s partner; stepmother/father’s partner; aunt/uncle/cousin; grandparent; other extended family; other*)
First abuse type	Type of abuse first disclosed by texter	Categorical (*physical; sexual; emotional/psychological; neglect*)
All abuse types	All abuse types disclosed by texter	Multiple-selection categorical (*physical; sexual; emotional/psychological; neglect*)
Length of victimization	Length of abuse victimization reported by youth	Categorical (*acute: first time/only time; chronic: multiple times*)
Help-seeking	Text of reason why youth reached out at this point rather than previously or at a point in the future	Open response
Other issues	Other nonabuse issues disclosed by the texter	Multiple-selection categorical (*bullying; eating disorder; friend issues; sexual or gender-identity issues; mental health; school problems; self-harm; substance abuse; suicidal thoughts/ideation; suicide attempt*)
Other interactions	Text of other interactions that were particularly representative of themes	Open response

## Results

### Overview

A total of 244 conversations from 236 individuals resulted in a mandatory report. The average age of texters was 14.3 years (SD 1.8, range 7-17). In 29 out of 244 conversations (11.9%), the specific age of the user was not confirmed, but the texter was a minor (eg, reported they were under 18). Gender identity was rarely explicitly discussed by the crisis counselor or the user.

The texters often discussed other psychosocial issues beyond the maltreatment and many reported concurrent issues. Nearly a quarter of conversations (55/244, 22.5%) reported suicidal desire, 16.4% (40/244) reported having access to the intended lethal means for suicide, and 10.2% (25/244) reported having a timeline for the suicide. Mental health issues, including depression (29/244, 11.9%), self-harm (29/244, 11.9%), anxiety (22/244, 9.0%), and stress (21/244, 8.6%) were also commonly reported by texters.

### Focus of Initial Texts With the Crisis Counselor

Upon receiving a message from a texter, the crisis service platform automatically issues a text asking for additional information. In response to the initial query, nearly half of texters discussed abuse (see Table 2). Many of these initial disclosures included a variant of the word *abuse*, such as “I think my parents are abusive...” or “I’ve been having problems with my mom with abuse and neglect.” Other initial disclosures included a description of the situation, for example, “...he [father] tried to swing a broken bottle at my head !!” and “My brother just beat me. he’s 18.”

Other responses to the initial, automated query focused on a range of other issues. General family issues, such as “I hate my dad,” “I dont want to be with my family no more...,” and “My dad is freaking me the hell out...,” were the most common initial responses. Disclosures of suicide and self-harm (eg, “I feel like not living anymore”) or advice and support-seeking (eg, “I need help”) were also common in these initial texts. A few other responses focused on mental health, running away, or other topics.

### Characteristics of Disclosed Abuse

The most common perpetrator of abuse disclosed by texters were parents (mom, 121/244, 49.6%; dad, 113/244, 46.3%). Stepfathers/mother’s partners (18/244, 7.4%), brothers (16/244, 6.6%), and grandparents (14/244, 5.7%) were also frequently mentioned. Physical abuse was the most common form of abuse discussed in the initial abuse disclosure (106/244, 43.4%). Emotional or psychological abuse (83/244, 34.0%), sexual abuse (38/244, 15.6%), and neglect (15/244, 6.1%) were also included. Many texters discussed multiple types of abuse. Nearly three-quarters of texters talked about physical abuse (173/244, 70.9%) and more than half discussed emotional or psychological abuse (138/244, 56.6%). Sexual abuse (51/244, 20.9%) and neglect (26/244, 10.7%) were less common. Texters reported, on average, 1.59 (SD 0.6) types of abuse victimization.

**Table 2:** Distribution of content of initial message and first maltreatment disclosure (N=244).

Theme		n (%)	Example quote
**Initial message**			
	Disclosure of abuse	108 (44.3)	“My dads abusing me and I have no escape”
	Family issues	33 (13.5)	“I dont want to be with my family no more im tired of being in this family”
	Suicide/self-harm	25 (10.2)	“i feel extremely alone and I honestly want to die”
	General support-seeking	15 (6.1)	“I need someone to talk to”
	Limited information	15 (6.1)	“A lot of things”
	Other mental health	5 (2.0)	“Just really depressed is all”
	Running away	5 (2.0)	“i am thinking about running away. i think i will run away...”
	Other	38 (15.6)	“I want to stop being hurt”
**First maltreatment disclosure**			
	Description of event consistent with abuse	119 (48.8)	“touched me in a place I didn’t want to be touched”
	Abuse	99 (40.6)	“my parents are abusive”
	Other definite language	16 (6.6)	“father rapes me...”
	Other	6 (2.5)	“my mom enjoys punishing me”
	No identifiable child maltreatment disclosure	4 (1.6)	N/A^a^

^a^N/A: not applicable.

For many texters, their abuse experiences were chronic. Of the 221 conversations where chronicity of abuse was discussed, 92.8% (205/221) of texters discussed recurrent abuse. A recent crisis or escalation in the abuse was a common reason for texters to initiate contact. Several texters discussed issues with divorce or custody arrangements involving an impending visit with the noncustodial, abusive parent. Some texters discussed an increase in the frequency or severity of the abuse prior to reaching out for help. Other texters disclosed they had recently unsuccessfully attempted to reach out for help from extended family or adults in the community and were seeking support in managing the trauma associated with the abuse or assistance reporting the abuse.

### Language Used in Maltreatment Disclosure

In their initial maltreatment disclosures, texters most commonly described experiences consistent with abuse without explicitly naming it as such (see Table 2). However, nearly half of texters included a variant of the word *abuse* in their initial disclosure of child maltreatment (eg, *abusive, abused*, or *abuse*) or other definite language (eg, *raped, molested*, or *assaulted*). Phrases related to hitting (eg, “mom hit me” or “he hits me”) were frequently used, as was language related to being beaten (eg, “beat me” or “get beat up”) and being forced to engage in sexual touching (eg, “forced me to have sex” or “touched me in my sleep”). Other participants described very specific incidents (eg, “say she will burn the house down with me in it” or “threatened to pull a gun on me”). In a few instances, crisis counselors asked participants if they were being abused or clarified the seriousness of the issues, as it was not clear if the user perceived the behavior to be irritating or truly harmful.

### Language Used in Comparison Cases

In the 50 randomly selected conversations that included any of the terms commonly used during disclosure (ie, *abuse, abused, abusive*, or *molested*) from the overall pool of messages exchanged within the platform, the research team found no indication that texters use the word *abuse* to describe nonabuse situations; however, these words did not always indicate recent child abuse where mandatory reporting would have been appropriate. In some instances, texters were referring to intimate partner violence or substance abuse, which do not fall under the jurisdiction of child maltreatment mandatory reporting. In others, the texters inquired about the confidentiality policy of the crisis service and the crisis counselors detailed the instances, including abuse, when confidentiality could be broken.

In the conversations in which the abuse-related words were used to describe child maltreatment, the disclosure and details about child maltreatment in this additional sample were quite like the disclosures that resulted in a mandatory report. They included vivid descriptions of the abuse, such as “My stepdad beat the shit out of me...,” “He held a knife to my stomach. He told me if he wanted he could kill me in an instant...,” and “She [sister] pushed me then proceeded to grab my hair, and throaty then attempt to punch me.” There were two main reasons why these conversations were not reported through the mandatory reporting process. First, more than half of the texters were discussing historical events, such as an adult saying, “I was sexually abused when I was 14-15 years old...” or a child saying, “I got molested by my dad for four years, TWO YEARS AGO.” In other instances, the texter withheld key information necessary to make a report (eg, name and location). The crisis service does not retain identifiable information of the texter; it is only available if the texter agrees to provide it.

In other conversations, texters withheld their identifying information because they were concerned about the consequences of mandatory reporting. In some situations, texters had prior experience with child protective services and decided the costs of re-engaging with the system (eg, “and the caseworker hasn’t done anything about it yet...”) were outweighed by the potential benefits. Some texters had fears related to parents finding out about the disclosure (eg, “My parents can’t kbiw about this” or “No That [stepfather finding out about disclosure] would be worse”).

## Discussion

### Principal Findings

Disclosure of child maltreatment is a complex and difficult process for many young victims but is a critical step to receiving support that would ideally end the maltreatment. Technology-based approaches may represent a novel method for young people to seek support [9], and this study suggests young people disclose their experiences through SMS-based services. More than half of texters whose conversation resulted in a mandatory report discussed abuse or other significant family issues in the first response. Texters were also explicit in their initial disclosure language, with almost all cases including the word *abuse*, other definite language indicating assault, or an explicit description of the experience. Early disclosure, combined with explicit language, may suggest at least a portion of young victims are actively seeking safe ways to talk about their experiences, rather than incidentally sharing experiences while seeking support for other issues.

As SMS-based and other brief written communication-based crisis services (eg, online chat and forums) expand, it will be vital to collaboratively develop and expand evidence-based best practices and trainings with providers to ensure texters have a positive disclosure experience. Although there is limited research focused on disclosure of physical and emotional child abuse and neglect [3], there is considerable research on disclosure of sexual abuse and assault in both children and adults. Sexual assault experiences are unlikely to be directly analogous to child maltreatment experience but may share some similarities in the disclosure process; therefore, the sexual assault research literature may provide guidance on easing the disclosure process for young people who have experienced child maltreatment [1,2,19]. Authors of a study on sexual assault disclosure in an online forum found that individuals were often directly seeking information, network, or emotional support [20]. In general, people seeking informational and emotional support were more likely to post these requests anonymously, which the authors theorized was due to fear of unsupportive responses [20]. These fears may be justified, as another study found that while most disclosures in an online forum received positive responses, several responses were categorized as blaming, doubting, or being generally unsupportive [21]. Social responses to sexual assault disclosure have been strongly associated with postassault mental health and well-being [22,23], and it is likely the relationship between social responses to disclosure and well-being persist for child maltreatment victims. As a result, organizations providing this type of service may need to have a moderation process for interactions between users, if applicable, and train affiliated staff or volunteers to appropriately respond to disclosure to ensure young people receive an appropriate response.

### Implications

There is considerable evidence to suggest that adolescents and young adults prefer brief written communication to verbal communication [9]. Since young people have readily adopted SMS-based communication, it may be prudent for other social and health services who engage young users around sensitive information to implement brief written communication options. For example, child welfare services could explore adding an SMS or online chat reporting option, which could encourage young people to disclose their own experiences and connect them to individuals specifically trained in child maltreatment. However, additional research must be conducted to determine the feasibility, acceptability, and effectiveness of use. If acceptable and effective, these strategies must be judiciously and deliberately implemented to ensure a positive experience for the user.

Additional research in this area must continue to carefully consider the ethical implications of using technology-based data created for purposes other than research. The platform used in this study disclosed on their website that data may be shared with external research partners to support research, policy, and community organizing. In addition, users may request to have their data removed from the database. The platform also created an independent data ethics committee, based their research vetting practices on other established data warehouses; they auto-scrubbed data of identifying information and required researchers to work in a restricted data enclave.

However, recent controversies surrounding Internet privacy may necessitate that researchers take additional precautions when conducting research using technology-based data created for purposes other than research [24]. The Association of Internet Researchers suggests that researchers carefully consider several guidelines prior to using technology-based data, including the users’ expectations around privacy and who may benefit or be harmed from the study [25,26]. Due to the sensitive nature of this data, users would likely be embarrassed, hurt, or angry if their expectations of data privacy did not match those of the service and the researchers. In partnership with the platform used in this study, we took precautions to ensure that users could not be indirectly identified through our research outputs. We carefully selected quotes that were general enough to apply to many situations and would not be recognizable as uniquely attributable to a specific user or situation. We, and other researchers in this area, will need to continue to critically consider ethical approaches to Internet and technology-driven research as technology, data management, and privacy standards evolve.

### Limitations

Given the scale of the crisis service data, it was necessary to focus on a segment of the data for this first research study. As a result, the primary dataset was restricted to conversations resulting in mandatory report with limited exploration of other conversations for sensitivity analysis. The results may not generalize to other types of conversations, as they represent the users willing to allow crisis counselors to seek services on their behalf. Users most fearful of the perpetrator and users least troubled by their victimization may be less willing to take that step. It may be beneficial to engage with computer scientists to develop machine learning algorithms that would allow processing of the entire dataset and that may improve the sensitivity and specificity of screening.

In addition, only one researcher had access to the raw conversation files as part of the data-sharing agreement, so only she could code the conversations. Her training and experience in child maltreatment research likely influenced some aspects of the research, such as which segment of the conversation constituted the initial disclosure of abuse. However, the researcher also has training in qualitative methods and was intentionally mindful of how this training may influence her coding.

The crisis service may also represent a unique environment for disclosure, as it is user-directed and anonymous. Users may feel more secure sharing explicit details of their experiences because they are able to disengage at any point and are not personally identifiable. Additional evaluation of the suitability of SMS approaches in personally identifiable situations is necessary.

### Conclusions

This study found that young people are seeking support related to child abuse in an SMS-based crisis service. As additional SMS- and technology-based approaches develop, it may be beneficial to evaluate how these methods of communication may be built into systems that regularly engage with young people. However, it will be necessary to explicitly consider how to address child maltreatment disclosures within the systems. In addition, careful consideration of evaluation methods for these approaches and the ethical use of data created by these systems will be necessary. If feasible and acceptable methods are developed and evaluated, SMS or other brief written communication platforms may improve the communication between young people and health and human service organizations.

## Abbreviations

CTL: Crisis Text Line
SMS: short message service
